# PD-L1 regulates c-MET phosphorylation and contributes to MET-dependent resistance to osimertinib in EGFR-mutant NSCLC

**DOI:** 10.1186/s12929-025-01181-3

**Published:** 2025-10-09

**Authors:** Chia-Chi Hsu, Derek De-Rui Huang, Wei-Hsun Hsu, Min-Shu Hsieh, Fang-Yu Lin, Pei-Chen Tai, Shih-Yun Chou, Hsin-Hui Tsai, Chen-Yuan Tseng, Bin-Chi Liao, Jin-Yuan Shih, James Chih-Hsin Yang

**Affiliations:** 1 Department of Oncology, National Taiwan University Hospital, Taipei, Taiwan; 2 Graduate Institute of Oncology, National Taiwan University, Taipei, Taiwan; 3 Department of Medical Oncology, National Taiwan University Cancer Center, National Taiwan University Hospital, No.57, Ln. 155, Sec. 3, Keelung Rd., Da’an Dist., 106 Taipei, Taiwan; 4 Department of Pathology and Graduate Institute of Pathology, National Taiwan University, Taipei, Taiwan; 5 Department of Pathology, National Taiwan University Cancer Center, National Taiwan University Hospital, Taipei, Taiwan; 6 Institute of Molecular Biology, National Chung Hsing University, Taichung, Taiwan; 7 Department of Internal Medicine, National Taiwan University Hospital, Taipei, Taiwan; 8 Cancer Biology Research Group, Center of Precision Medicine, National Taiwan University, Taipei, Taiwan

**Keywords:** Non-small cell lung cancer, Epidermal growth factor receptor tyrosine kinase inhibitor, Osimertinib resistance, Programmed death-ligand 1, *MET* amplification

## Abstract

**Background:**

Programmed death-ligand 1 (PD-L1) is a well-recognized predictive biomarker for immunotherapy in non-oncogene-addicted non-small cell lung cancer (NSCLC). However, its role in epidermal growth factor receptor (*EGFR*)-mutant NSCLC remains unclear. This study aims to investigate the impact of PD-L1 on the signaling pathways in *EGFR*-mutant NSCLC.

**Methods:**

The regulatory role of PD-L1 was investigated through in vitro manipulation of PD-L1 expression across several EGFR-mutant cell lines, followed by analysis via human receptor tyrosine kinase (RTK) array, Western blotting, protein tyrosine phosphatase (PTPs) activity assays, and mRNA expression profiling. In vivo experiments were carried out using xenograft mice implanted with parental, PD-L1 knock-out and PD-L1 overexpression NCI-H1975 cells. Osimertinib was orally administered to the mice until tumor progression to evaluate the impact of PD-L1 on osimertinib resistance.

**Results:**

In human RTK array screening, c-MET phosphorylation was found to be increased in EGFR-mutant PD-L1 overexpressing cells. We found that PD-L1 overexpression upregulated c-MET phosphorylation, while PD-L1 knock-out and knock-down resulted in downregulation of c-MET phosphorylation. Furthermore, we showed that PD-L1 upregulates c-MET phosphorylation by suppressing PTP activity and reducing mRNA expression in selected PTPs. In xenograft mice, *MET* amplification only developed in PD-L1 overexpression, but not in PD-L1 knock-out and parental NCI-H1975 cells, at the time of osimertinib resistance.

**Conclusion:**

In *EGFR*-mutant NSCLC, PD-L1 regulates c-MET phosphorylation and promotes *MET* amplification, contributing to osimertinib resistance.

**Supplementary Information:**

The online version contains supplementary material available at 10.1186/s12929-025-01181-3.

## Background

The aberrant activation of epidermal growth factor receptor (EGFR) tyrosine kinase domain due to *EGFR* gene mutation is a well-established mechanism leading to non-small cell lung cancer (NSCLC), affecting 40%–60% of East Asian patients or 10%–20% of Caucasian patients with lung adenocarcinomas [1, 2]. In *EGFR*-mutant NSCLC patients, targeted therapy with EGFR tyrosine kinase inhibitors (EGFR-TKIs) has brought remarkable treatment efficacy characterized by rapid disease control and durable responses [3]. With the introduction of the third-generation EGFR-TKI osimertinib as a first-line treatment option, the median progression-free survival for patients with advanced *EGFR* mutation NSCLC now exceeds 18 months [4, 5].

Programmed death ligand-1 (PD-L1), expressed in 40–50% of *EGFR*-mutant NSCLC tumors, is a key immune checkpoint that binds to its receptor PD-1 on T cells to enable tumor cells to evade immune surveillance and proliferate unchecked [6, 7]. Beyond its role as an immune checkpoint, PD-L1 has also been shown to possess direct signal transduction capacity that promotes the development of treatment resistance [8–11]. While PD-L1 is an important predictive biomarker for immune checkpoint inhibitors (ICIs) in NSCLC patients without actionable genomic alterations [12–14], PD-L1 expression level does not consistently predict the efficacy of ICIs in *EGFR*-mutant NSCLC patients [15–17]. Several retrospective clinical studies suggest that *EGFR-*mutant NSCLC patients with baseline tumor PD-L1 expression greater than 50% are associated with lower response rate and earlier development of treatment resistance to EGFR-TKI [18–20]. On the other hand, a biomarker analysis from a subset of patients in the FLAURA trial showed that first-line osimertinib’s efficacy was not significantly different based on PD-L1 expression when using a cut-off of 1% [21].

In *EGFR*-mutant NSCLC treated with osimertinib, the most common resistance mechanism is the acquired amplification of the *MET* proto-oncogene, occurring in approximately 15–20% of patients [22–24]. Unfortunately, no clinically reliable predictive biomarker for *MET* amplification exists to date. In a small EGFR-TKI resistant cohort, PD-L1 expression has been positively correlated with c-MET expression in post-progression tumor specimens immunohistochemically [10]. Preclinical investigations using NSCLC cell lines harboring *EGFR* mutation have shown that *MET* amplification has the potential to alter PD-L1 expression in EGFR-TKI resistant cells [10]. However, whether PD-L1 can regulate c-MET pathway remains largely unexplored.

This study aims to investigate the role of PD-L1 in *EGFR*-mutant NSCLC and its contribution to EGFR-TKI resistance. We first discovered that the overexpression of PD-L1 led to upregulation of c-MET phosphorylation. Subsequently, we determined that PD-L1 regulates c-MET phosphorylation via a protein tyrosine phosphatase (PTP)-dependent pathway. Furthermore, through in vivo experiments, we reveal that *EGFR*-mutant NSCLC cells with PD-L1 overexpression are predisposed to develop *MET*-amplification following osimertinib treatment.

## Methods

### Reagents and antibodies

Osimertinib (AZD9291) was provided by AstraZeneca Pharmaceuticals. Capmatinib (Cat. No. S2788) and tepotinib (Cat. No. S7067) were purchased from Selleckchem. Sodium orthovanadate (Na_3_VO_4_) (Cat. No. S6508) and Bis-Maltolato-OxoVanadium (IV) (BMOV) (Cat. No. SML1079) were from Sigma-Aldrich. Osimertinib, capmatinib, tepotinib, and BMOV were dissolved in DMSO at a stock concentration of 10 mM and stored at −80 °C. Na_3_VO_4_ was dissolved in H_2_O at 0.5 M and stored at −20 °C. Anti-PD-L1 monoclonal antibodies, durvalumab and atezolizumab, were purchased from Selleckchem. Restriction enzymes were purchased from New England Biolabs. Antibodies against PD-L1, phospho-c-MET (Y1234/Y1235), phospho-c-MET (Y1349), and c-MET were from Cell Signaling Technologies. Antibodies against α-tubulin was from Sigma-Aldrich.

### Cell cultures

Human *EGFR*-mutant NSCLC lines (HCC4006, HCC827, NCI-H1650, NCI-H1975 and PC9) were maintained in RPMI-1640 with 10% FBS, 2 mM L-glutamine, and antibiotic (100 units/ mL penicillin G, 100 µg/ mL streptomycin sulfate, and 250 ng/ mL amphotericin B). Cells were incubated in a humidified atmosphere of 5% CO_2_ at 37 °C.

### Transfection and generation of PD-L1 stable clones

Transfection experiment was conducted as previously described [25]. Cells were grown to approximately 70% confluence for transfection. PD-L1 plasmids (Origene) were transfected into cells using the Lipofectamine 2000 (Invitrogen) transfection reagent according to the manufacturer’s instructions. After 48 h, G418 (600 µg/mL) was added to select stable clones.

### Receptor tyrosine kinase (RTK) array

Human Phosphor-Receptor Tyrosine Kinase Array Kit (R&D Systems) was used to detect phosphorylation of 49 RTKs. Briefly, the array membranes were blocked by Array Buffer 1 for 1 h at 24 °C, and incubated with whole cell lysates (200 µg) prepared by Array Lysis Buffer 17 overnight at 4 °C on a rocking platform shaker. The array membranes were then washed three times by washing buffer, and were incubated with Anti-Phosphor-Tyrosine-HRP Detection Antibody for 2 h at 24 °C. The array membranes were then developed using Chemi Reagent Mixture, and kinase intensities were analyzed using Image-J software.

### Western blot analysis

The western blot analysis was conducted by the protocol of previous study. Whole cell lysates were prepared using RIPA buffer (Cell Signaling Technologies) and phosphatase inhibitors. Protein concentrations were determined by Bradford assay. Samples were diluted in 5X Laemmli buffer (300 mM Tris–HCl pH 6.8, 10% SDS (w/v), 5%, 2-mercaptoethanol, and 25% glycerol (v/v), 0.1% bromophenol blue w/v) and boiled for 5 min. Twenty micrograms of proteins were separated by 8–15% SDS-PAGE and transferred onto polyvinylidene fluoride (PVDF) membranes (GE Healthcare Life Sciences). Nonspecific binding sites on the PVDF membranes were blocked with 5% non-fat milk in TBST (20 mM Tris–HCl, pH 7.6, 137 mM NaCl, 1% Tween-20). Membranes were hybridized with primary antibodies overnight at 4 °C, followed by incubation with horseradish peroxidase (HRP)-conjugated secondary antibodies for 1 h at 24 °C. Protein bands were developed using Immobilon western chemiluminescence substrates.

### Establishment of PD-L1 knock-out (KO) lines using CRISPR system

sgRNAs were designed using the Guide-Design-Resources tool (https://zlab.bio/guide-design-resources), and cloned into CRISPR plasmid (pSpCas9(BB)-2A-Puro, Addgene) by *BbsI* sites. Plasmids were transfected into HEK293T cells, and the sgRNA activity was assessed using Guide-it Mutation Detection Kit (Takara Bio) according to the manufacturer’s instruction. The plasmid encoding sgRNA with best activity against PD-L1 (sgRNA#1) was transfected into *EGFR*-mutant NSCLC cells using transfection protocol mentioned above. Puromycin (1 μg/mL) was used for selection, and single cell clones were expanded and screened for PD-L1 knockout.

### Small-interfering RNA (siRNA) knock-down

The siRNA against PD-L1 and the cherry-pick custom library siRNAs against protein tyrosine phosphatase (PTP) family were purchased from Horizon Discovery, and were transfected into target cells using Lipofectamine^™^ RNAiMAX Transfection Reagent (Thermo Fisher Scientific) according to the manufacturer’s instructions. After 48 h of transfection, the efficiency of knock-down and the effects of siRNA against PTP family on phospho-c-MET were validated using Western blot analysis.

### HGF gene expression analysis using EMBL-EBI expression Atlas

To investigate the gene expression of HGF in human lung adenocarcinoma (LUAD), we queried the EMBL-EBI Expression Atlas database [26], which provides curated transcriptomic datasets across various cancer types. We focused on the E-MTAB-2706 dataset [27], which contains RNA-seq data from a broad cohort of tumor samples, including LUAD. Transcripts per million (TPM) values for the HGF gene were extracted and analyzed to assess its baseline expression levels in LUAD tissues. These values were used for comparative visualization and interpretation alongside our experimental data.

### Secreted human hepatocyte growth factor (HGF) ELISA assay

To quantify secreted hepatocyte growth factor (HGF) in culture supernatants, we utilized the Human HGF ELISA Kit (abcam) according to the manufacturer’s instructions with minor modifications. Cells were seeded at a density of 3 × 10^5^ cells per well in 6-well plates and maintained under standard culture conditions until adherent. Once adherence was confirmed, cells were gently washed twice with serum-free RPMI medium and incubated in 3 mL of serum-free RPMI for 24 h. After incubation, 1 mL of conditioned medium was collected from each well. Samples were either immediately processed or centrifuged to remove debris and stored at −80 °C until analysis. For ELISA, 50 μL of sample or standard was added to each well of the pre-coated 96-well plate, followed by 50 μL of antibody cocktail. Plates were incubated at room temperature for 1 h on a shaker (400 rpm). Wells were then washed three times with 350 μL of 1X Wash Buffer PT, and 100 μL of TMB Development Solution was added to each well and incubated for 10 min in the dark. The reaction was terminated with 100 μL of Stop Solution, and absorbance was measured at 450 nm using a microplate reader. All samples were analyzed in duplicate, and HGF concentrations were calculated based on the standard curve.

### Protein tyrosine phosphatase (PTP) activity assay

The measurement of PTP activity was carried out using PTP assay kit (abcam) according to the manufacturer’s instruction. Briefly, the cell pellet was resuspended with PTPase assay buffer containing 2 mM dithiothreitol (DTT) and cocktail of protease inhibitor, followed by sonication under the conditions of 30% amplitude for 5 s on and 5 s off for a total of four cycles. The supernatant was obtained by centrifuging at 10,000xg, 4 °C, 15 min, and the was loaded into a 96-well black plate. After adding the PTPase substrate, fluorescence was immediately measured by an ELISA reader at Ex/Em = 368/460 nm in kinetic mode for 30–45 min at 25 °C.

### RNA sequencing

The purified RNA was used for the preparation of the sequencing library by TruSeq Stranded mRNA Library Prep Kit (Illumina, San Diego, CA, USA) following the manufacturer’s recommendations. Briefly, mRNA was purified from total RNA (1 μg) by oligo(dT)-coupled magnetic beads and fragmented into small pieces under elevated temperature. The first-strand cDNA was synthesized using reverse transcriptase and random primers. After the generation of double-strand cDNA and adenylation on 3’ ends of DNA fragments, the adaptors were ligated. The products were enriched with PCR and purified with AMPure XP system (Beckman Coulter, Beverly, USA). The libraries were qualified by Qsep400 System (Bioptic Inc., Taiwan) and quantified by Qubit 2.0 Fluorometer (Thermo Scientific, Waltham, MA, USA). The qualified libraries were then sequenced on an Illumina NovaSeq platform with 150 bp paired-end reads generated by Genomics, BioSci & Tech Co., New Taipei City, Taiwan.

### Cytotoxic assay

The sulforhodamine B (SRB) assay was performed to measure cell viability [28]. Cells (1 × 10^3^ cells/well) were seeded in 96-well plates in triplicates. After 96 h of drug treatment, cells were fixed using 10% ice-cold trichloroacetic acid (Sigma-Aldrich) at 4 °C for 1 h, rinsed 4 times with distilled water and air dried. The cells were then stained with 0.057% SRB (Sigma-Aldrich) in 1% acetic acid for 30 min at 24 °C. After washing 4 times with 1% acetic acid and air dried, 200 µL of 10 mM Tris-base (pH 10.5) was added into each well and incubated for 30 min at 24 °C. Absorbance was measured at 540 nm.

### Animal experiment

The experimental protocol for animal study was approved by the Institutional Animal Care and Use Committee of National Taiwan University and male NCr athymic nude mice (6 weeks of age) were purchased from the National Laboratory Animal Center. All animal experiments will be performed under specific pathogen free conditions in accordance with institutional guidelines. NCI-H1975 with stable over-expression of *CD274* (2 × 10^6^) were re-suspended in 100 µL serum-free RPMI-1640 and were subcutaneously injected into the rear flank of mice using a 30-gauge needle. The tumor volumes were determined using a digital caliper twice weekly, and were calculated using the following equation: volume (V) (mm^3^) = length × width^2^ /2. When tumor volume reached 0.5 cm^3^, animals were treated with osimertinib (2.5 mg/kg/day, oral gavage). Progression-free survival (PFS) was defined as the time from treatment initiation until the tumor volume reached 2 cm^3^. At this point, mice were sacrificed and the xenografted tumors were harvested for further analyses.

### *MET* copy number variation (CNV) TaqMan assay

*MET* CNV TaqMan assay was used to evaluate the relative *MET* copy number of testing samples. DNA was isolated from xenografted tumors or culture cells using NucleoSpin Tissue Kit (Macherey–Nagel), and was quantified by NanoPhotometer NP80 (IMPLEN) to 40 ng for experiments. The *MET* CNV was examined using MET TaqMan probe (Thermo Fisher Scientific, Hs01432482_cn), and the real-time PCR (RT-PCR) was performed using StepOnePlus Real-Time PCR System (Thermo Fisher Scientific). Data was normalized to the reference assay, RNase P.

### Statistical analysis

Data are presented as mean ± SEM. Statistical differences were determined using Student’s t-test, with p < 0.05 considered significant. Survival curves for progression-free survival (PFS) were generated for the three groups (Control, PD-L1 OE, PD-L1 KO), and statistical differences between the groups were determined using the Log-rank test. P-values are indicated on the survival curves in Fig. 5D.

## Results

### PD-L1 induces c-MET phosphorylation in human EGFR-mutant NSCLC cells

**Fig. 1:**
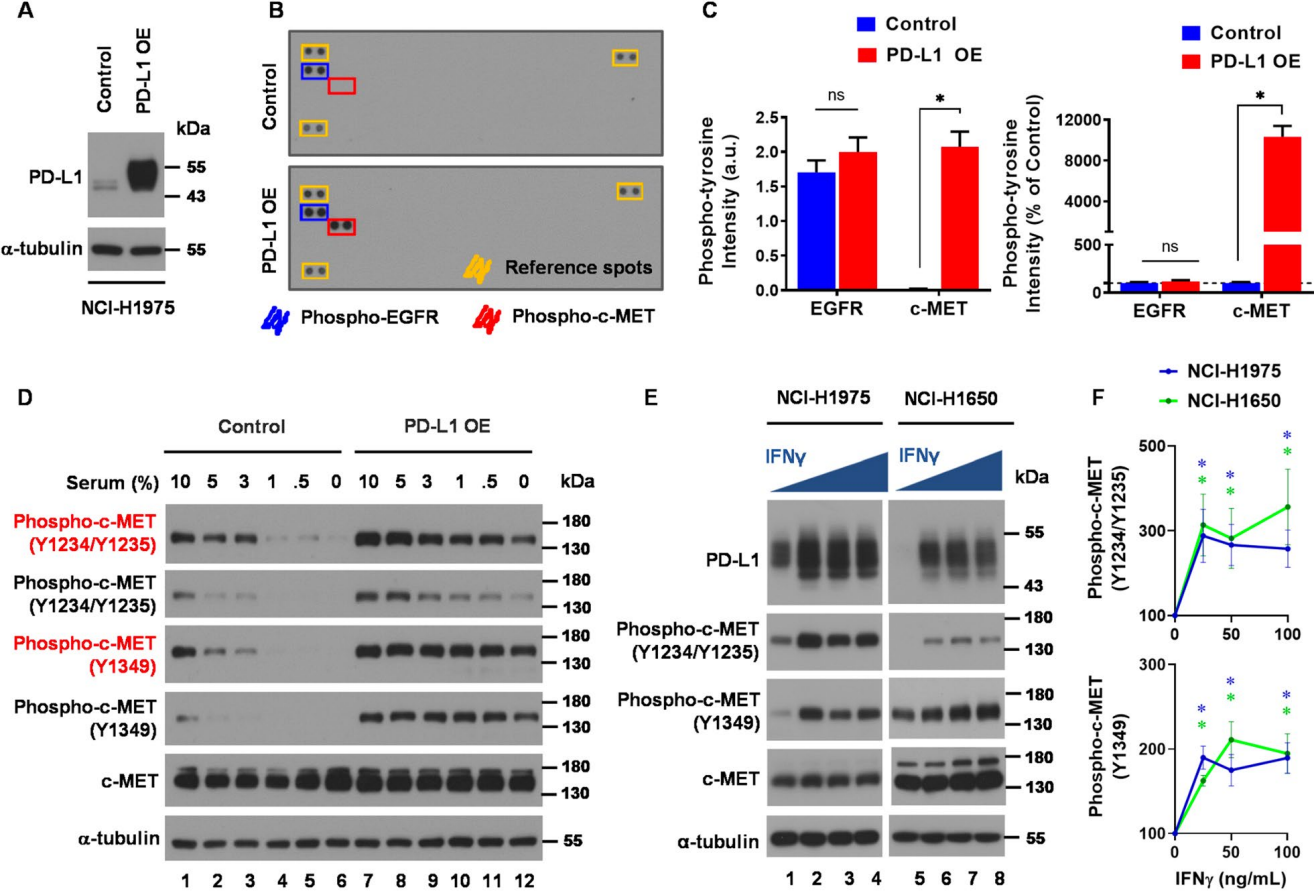
Induction of PD-L1 up-regulates c-MET phosphorylation in *EGFR*-mutant NSCLC cells. **A** Stable clones with exogenous expression of PD-L1 in NCI-H1975 cells. **B** and **C** Analysis of receptor tyrosine kinase in NCI-H1975 with PD-L1 overexpression. Bar graphs indicate the mean ± SEM. (*, p < 0.05). **D** Western blot analysis of phospho-c-MET expressions after incubation with different concentrations of serum (0, 0.5, 1, 3, 5, and 10%) for 24 h in NCI-H1975 with or without PD-L1 overexpression. **E** and** F** Western blot analysis of PD-L1 and phospho-c-MET expressions after IFN_γ_ (0, 25, 50, and 100 ng/mL) stimulation for 24 h in NCI-H1975 and NCI-1650 cells. Curve graphs indicate the mean ± SEM from three independent experiments. (*, p < 0.05)

To better understand the impact of PD-L1 expression on intracellular signaling in human *EGFR*-mutant NSCLC cells, we first utilized NCI-H1975 cells (EGFR L858R/T790M mutation) to establish stable overexpression of PD-L1 (PD-L1-OE) as depicted in Fig. 1A. Human receptor tyrosine kinase (RTK) array analysis of these NCI-H1975 PD-L1-OE cells revealed a significant increase of c-MET phosphorylation compared to NCI-H1975 control cells (Fig. 1B and C), even under serum-stress conditions (Fig. 1D and Supplementary. Figure 1). To further explore the influence of PD-L1 expression, we conducted interferon-gamma (IFNγ) stimulation experiments to examine the effect of endogenous PD-L1 expression on NCI-H1975 and NCI-H1650 cells (EGFR exon 19 del. E746-A750). After IFNγ stimulation, we observed a significant increase of c-MET phosphorylation following the induction of PD-L1 (Fig. 1E, lane 1–4 and Fig. 1F, upper panel for NCI-H1975; Fig. 1E, lane 5–8 and Fig. 1F, lower panel for NCI-H1650). These results suggested that PD-L1 overexpression led to upregulated c-MET phosphorylation.

**Fig. 2:**
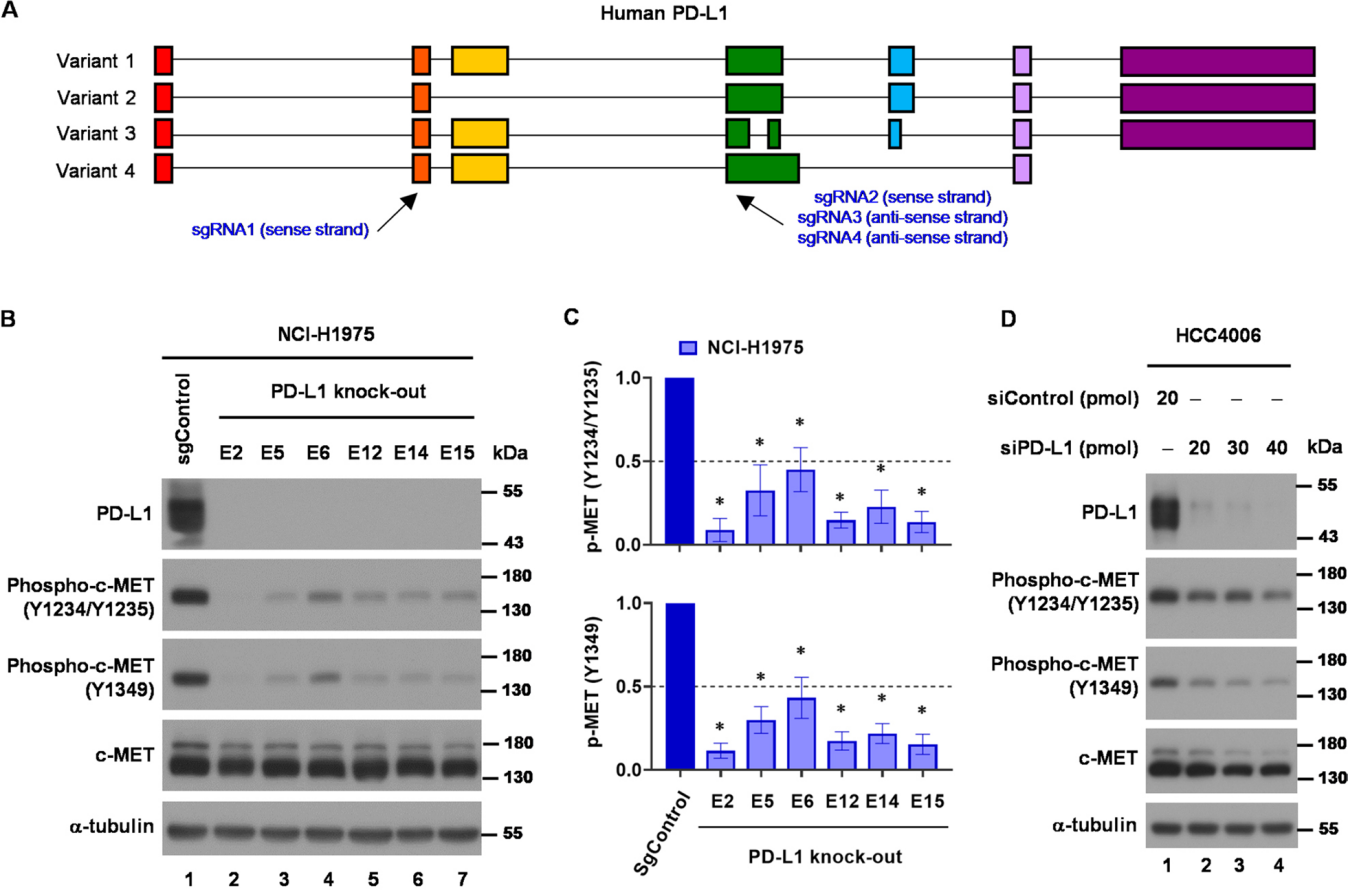
Genetic depletion of PD-L1 results in a decrease of c-MET phosphorylation in *EGFR*-mutant NSCLC cells. **A** Schematic representation of sgRNAs design and sequences for PD-L1 KO by CRISPR technology. **B** Western blot analysis of phospho-c-MET expressions after PD-L1 KO in NCI-H1975 cells. **C** Statistic analysis of Western blot analysis for phospho-c-MET (Y1234/Y1235) (upper) and phospho-c-MET (Y1349) (lower) after PD-L1 KO in NCI-H1975 cells. Bar graphs indicate the mean ± SEM. (*, p < 0.05). **D** Western blot analysis of phospho-c-MET expressions after knock-down of PD-L1 using siRNA technology in HCC4006 cells

Next, we aim to investigate the effect of reduced PD-L1 expression on c-MET phosphorylation. We designed four sgRNAs targeting all human PD-L1 variants for CRISPR knockout (KO) technology (Fig. 2A and Supplementary. Figure 2A), and select sgRNA#1 for subsequent KO experiments due to its favorable genomic editing activity (Supplementary Fig. 2B). The PD-L1 KO NCI-H1975 clones were generated and confirmed by Western blot (Supplementary Fig. 2C) and IFNγ stimulation experiments (Supplementary Fig. 2D). The c-MET phosphorylation significantly decreased in PD-L1 KO NCI-H1975 cells as compared to the control NCI-H1975 cells (Fig. 2B and C). The result was further supported by PD-L1 knockdown experiments using siRNA in NCI-H1975 (Supplementary Fig. 3) and HCC4006 cells (EGFR exon 19 del. L747-A750, P ins) (Fig. 2D), which also exhibited decreased c-MET phosphorylation following PD-L1 knockdown. These findings collectively indicated that c-MET phosphorylation could be regulated by PD-L1 expression in human *EGFR*-mutant NSCLC cells.

To further explore how PD-L1 regulates c-MET phosphorylation, we added anti-PD-L1 antibodies, atezolizumab and durvalumab, to NCI-H1975 PD-L1 OE cells to block the extracellular domain of PD-L1. In contrast to previous PD-L1 knock-out or knock-down experiments, c-MET phosphorylation was not reduced after adding anti-PD-L1 antibodies (Supplementary Fig. 4). Since atezolizumab and durvalumab act exclusively on the extracellular domain, this finding suggested that PD-L1 regulates c-MET phosphorylation through intracellular pathways independent of anti-PD-L1 blockade.

### PD-L1 upregulates c-MET phosphorylation by suppressing the activity of protein tyrosine phosphatases in human EGFR-mutant NSCLC cells

**Fig. 3:**
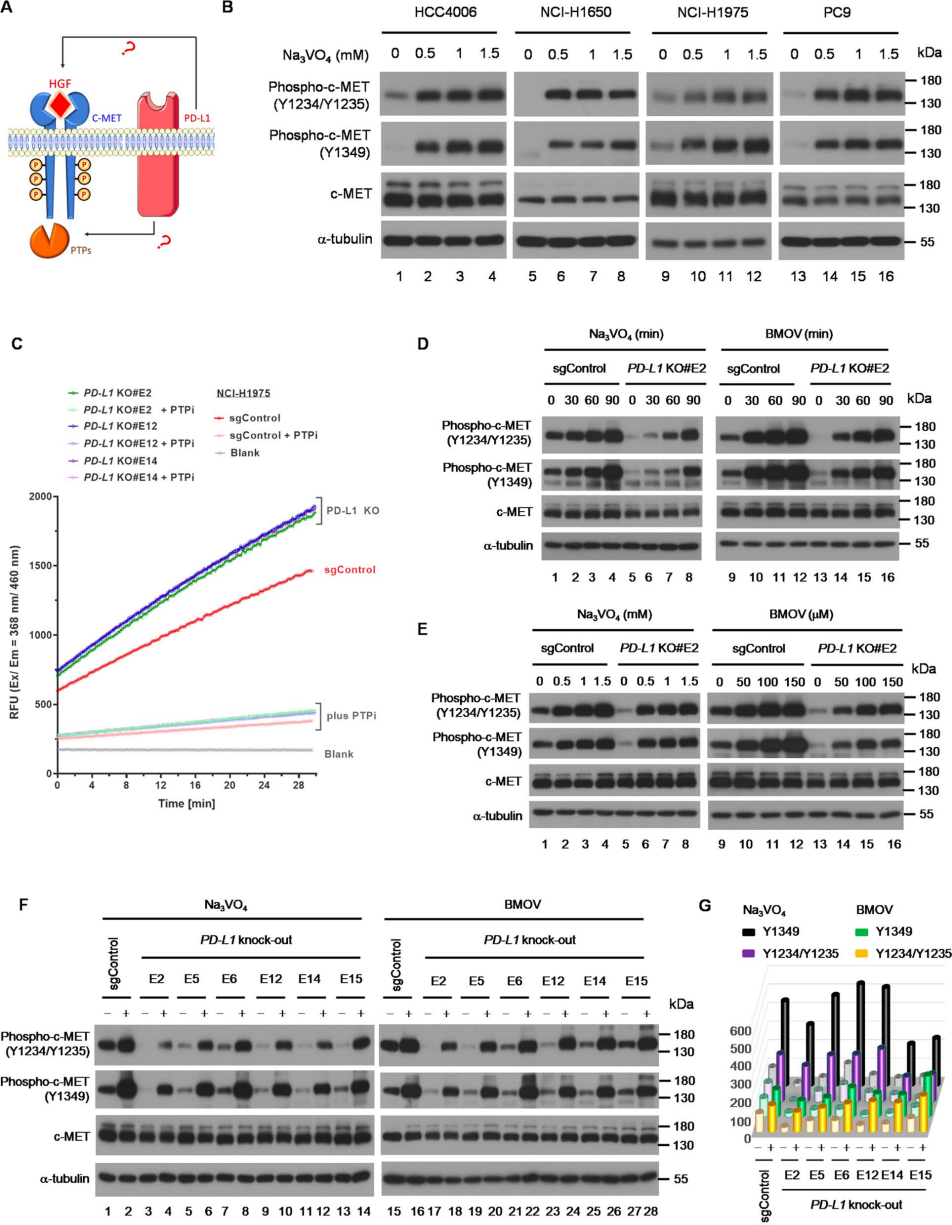
PD-L1 regulates the activity of protein tyrosine phosphatases, which contributes to the dephosphorylation of c-MET in human *EGFR*-mutant NSCLC cells. **A** Schematic representation of possible mechanism by which PD-L1 regulates c-MET phosphorylations. **B** Western blot analysis of phospho-c-MET expressions after treated with different concentrations of Na3VO4 (0, 0.5, 1, and 1.5 mM) for 90 min in HCC4006 (lane 1–4), NCI-H1650 (lane 5–8), NCI-H1975 (lane 9–12), and PC9 cells (lane 13–16). **C** Protein tyrosine phosphatase (PTP) activity assay in NCI-H1975 with or without PD-L1 KO. Western blot analysis of phospho-c-MET expressions in NCI-H1975 and PD-L1 KO E2 clone treated with Na3VO4 or BMOV in **D** time-course (0, 30, 60, and 90 min) (lane 1–8 for 1 mM Na3VO4; lane 9–16 for 100 μM BMOV) and **E** dose-dependent (lane 1-8for 0, 0.5, 1, and 1.5 mM Na3VO4 for 60 min; lane 9–16 for 0, 50, 100, and 150 μM BMOV for 60 min) experiments. **F** Western blot analysis of phospho-c-MET expressions in NCI-H1975 and all PD-L1 KO clones after treatment with 1 mM Na_3_VO_4_ (lane 1–14) or 100 μM BMOV (lane 15–28) for 90 min. **G** The bar chart represents the quantitative results from Western blot analysis

The phosphorylation of c-MET is dynamically controlled by auto-phosphorylation upon HGF binding and de-phosphorylation by protein tyrosine phosphatases (PTPs) [29]. Therefore, there are two potential hypotheses to explain why PD-L1 knockout leads to reduced c-MET phosphorylation in human *EGFR*-mutant NSCLC cells (Fig. 3A). Firstly, loss of PD-L1 expression leads to downregulation of HGF expression, hence downregulation of c-MET phosphorylation. Secondly, loss of PD-L1 expression leads to upregulation of PTPs activity, thereby enhancing the de-phosphorylation of c-MET, consequently causing downregulation of c-MET phosphorylation.

To answer this question, we first analyzed *HGF* expression in human lung adenocarcinoma (LUAD) using the data obtained from EMBL-EBI Expression Atlas Database [26]. The result showed that human *EGFR*-mutant cells barely express *HGF* at baseline, therefore it is unlikely that HGF expression will be further downregulated after PD-L1 KO (Supplementary Fig. 5A). Consistent with the database analysis, PD-L1 OE or KO did not significantly alter secreted HGF protein levels (Supplementary. Figure 5B and C). To investigate whether PTP affects c-MET phosphorylation, a known PTP inhibitor sodium orthovanadate (Na_3_VO_4_) was added to selected human *EGFR*-mutant NSCLC cells (Fig. 3B), including HCC4006 (lane 1–4), NCI-H1650 (lane 5–8), NCI-H1975 (lane 9–12), and PC9 (lane 13–16). The baseline c-MET phosphorylation markedly increased after adding Na_3_VO_4_, confirming PTP as a key regulator of c-MET phosphorylation. Enzyme kinetic assay was subsequently performed to directly evaluate PTP activity. In NCI-H1975 PD-L1 OE cells, the activity of PTP was reduced (Supplementary Fig. 6). Conversely, in NCI-H1975 PD-L1 KO cells, the activity of PTP was increased (Fig. 3C). These findings demonstrated that PTP activity can be regulated by PD-L1 in human *EGFR*-mutant NSCLC cells. Furthermore, inhibition of PTP by both Na_3_VO_4_ and Bis-Maltolato-OxoVanadium (IV) (BMOV, another pan-PTP inhibitor) successfully reversed the downregulation of c-MET phosphorylation observed in PD-L1 KO human *EGFR*-mutant NSCLC cells, as demonstrated by time-course (Fig. 3D) and dose-dependent (Fig. 3E) experiments. We also confirmed that downregulated c-MET phosphorylation could be restored after inhibition of PTP activity in all NCI-H1975 PD-L1 KO clones (Fig. 3F and G). In summary, these results suggested that PD-L1 upregulates c-MET phosphorylation by inhibiting the activity of PTPs.

### Multiple PTPs are involved in PD-L1 dependent c-MET phosphorylation

**Fig. 4:**
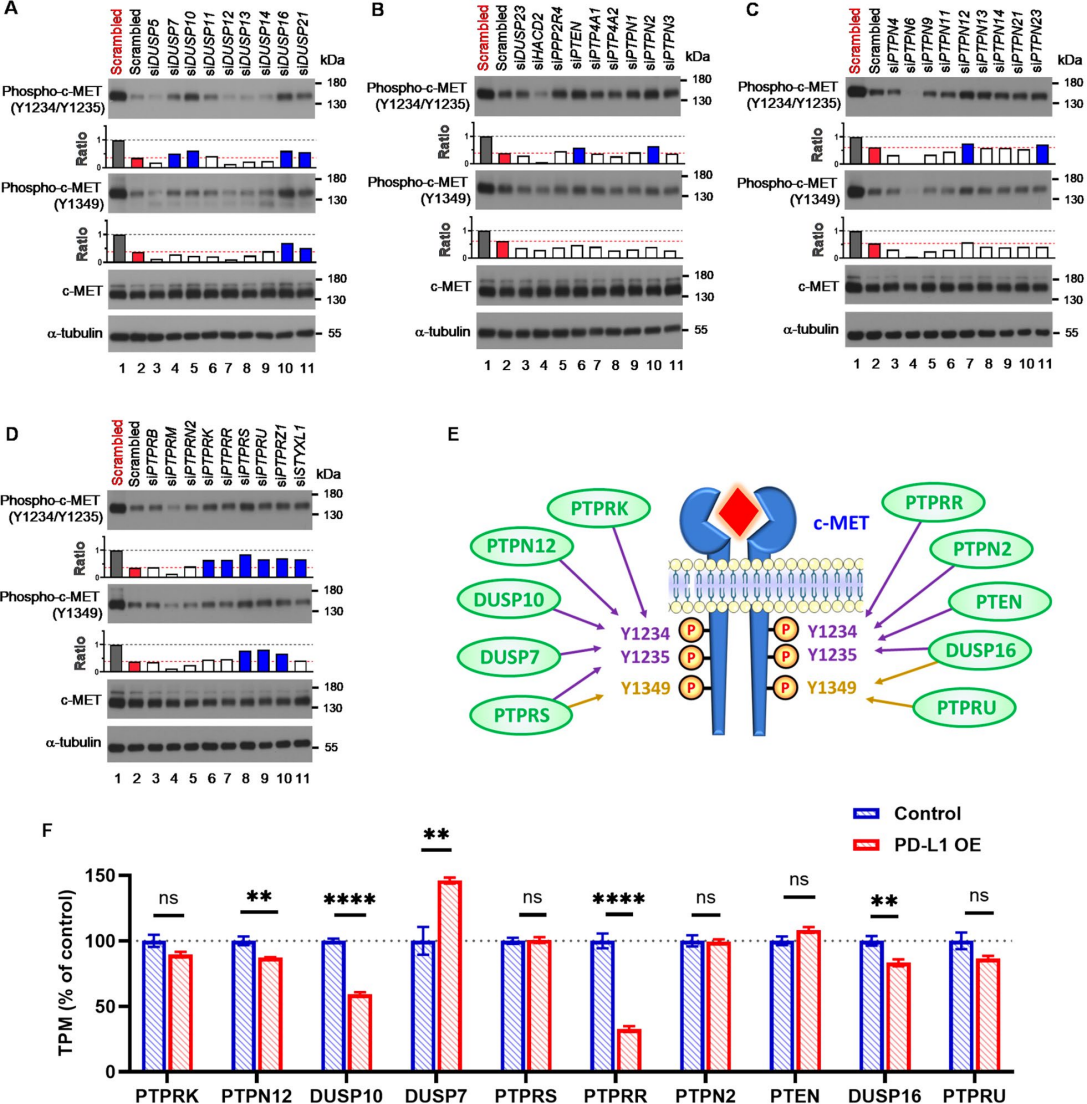
Multiple PTPs are involved in PD-L1 dependent c-MET phosphorylation. **A**–**D** The PTP members were individually knocked down in PD-L1 KO NCI-H1975 cells using transfection of siRNA. After transfection of 48 h, whole cell extract was harvested and the phosphor-c-MET as well as c-MET protein expression were analyzed using Western blot analysis. The bar charts represent the band intensities normalized to NCI-H1975 transfected with scrambled control (gray bar), while red bars represent normalized band intensities in PD-L1 KO NCI-H1975 cells. The bars changed from white to blue indicated that the down-regulation of phosphor-c-MET in PD-L1 KO NCI-H1975 cells was reversed knockdown of specific PTP members. **E** Schematic representation of the contribution of each PTP member to phosphorylations of c-MET in *EGFR*-mutant NSCLC cells. **F** The gene expressions of PTPRK, PTPN12, DUSP10, DUSP7, PTPRS, PTPRR, PTEN, DUSP16, and PTPRU in NCI-H1975 with (red bar) or without (blue bar) PD-L1 overexpression. *ns* non-significance; **, *P* < 0.01; ****, *P* < 0.001

The human PTP family controls the dynamic process of protein tyrosine phosphorylation. To investigate the role of each PTP family member in regulating c-MET phosphorylation in PD-L1 KO *EGFR*-mutant NSCLC cells, we used a custom library of siRNA to knock down the expression of each PTPs respectively (Supplementary Table 1). Among 36 PTPs, c-MET phosphorylation was partially restored after siRNA knock-down in ten PTPs, including *DUSP7*, *DUSP10*, *DUSP16*, *PTEN*, *PTPN2*, *PTPN12*, *PTPRK*, *PTPRR*, *PTPRS* and *PTPRU* respectively (Fig. 4A–D). These results indicated that these 10 PTPs were potential candidates involving in PD-L1 dependent c-MET phosphorylation (Fig. 4E). Subsequently, we compared the mRNA expression of these 10 PTPs between NCI-H1975 parental (control) and NCI-H1975-PD-L1-OE cells using RNA sequencing. Among these 10 PTPs, mRNA expression of *DUSP10*, *DUSP16*, *PTPN12* and *PTPRR* were significantly decreased in NCI-H1975-PD-L1-OE cells (Fig. 4F). These results showed that PD-L1 associated with multiple members of the PTP family rather than one specific PTP to regulate c-MET phosphorylation in EGFR-mutant NSCLC cells.

### EGFR-mutant NSCLC cells with PD-L1 overexpression and increased c-MET phosphorylation are predisposed to develop MET-dependent acquired resistance to osimertinib

**Fig. 5:**
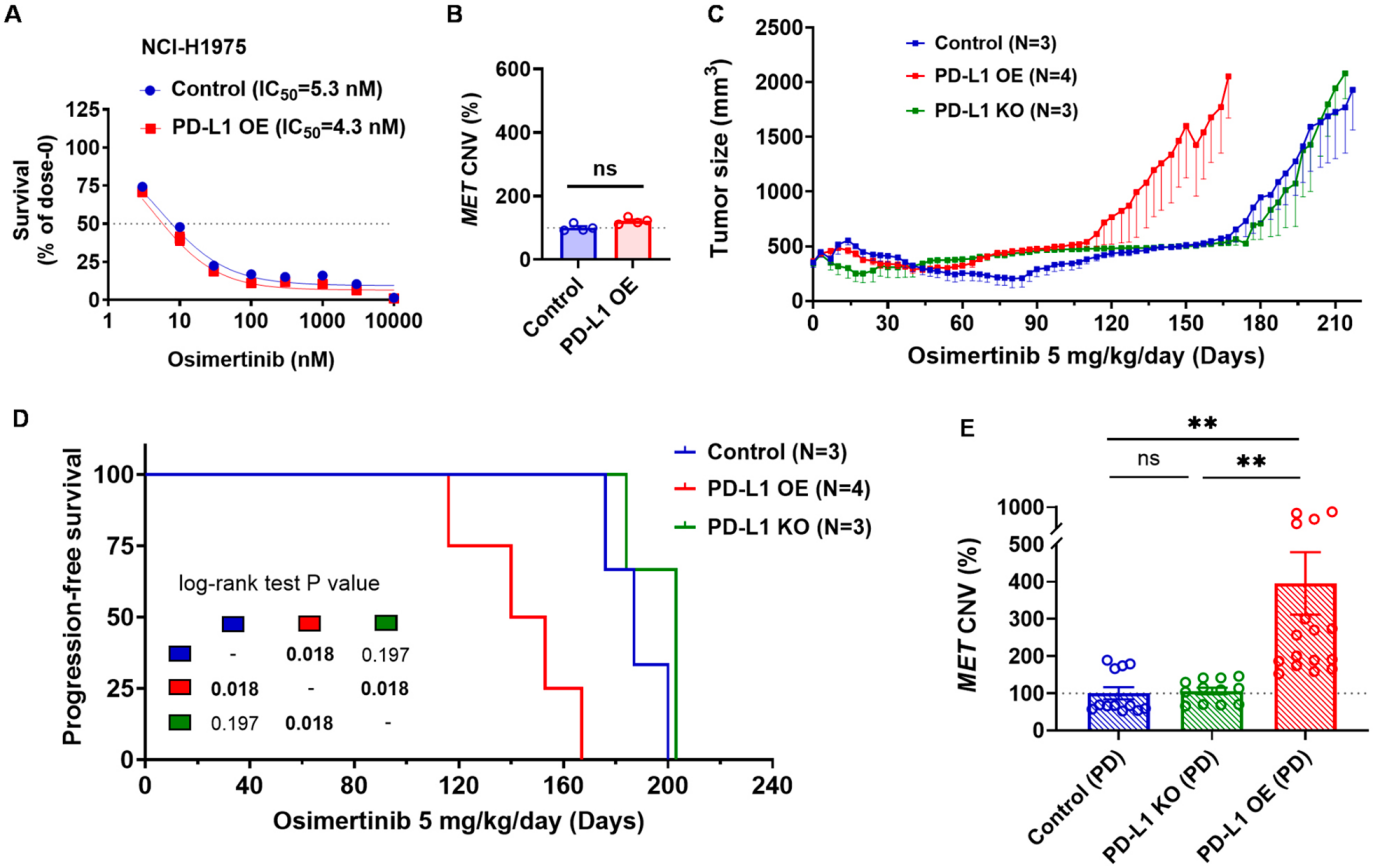
*EGFR*-mutant NSCLC cells with PD-L1 overexpression and increased c-MET phosphorylation are predisposed to develop MET-dependent acquired resistance to osimertinib. **A** The sensitivity to osimertinib treatment and the **B** MET CNV in NCI-H1975 cells with or without PD-L1 overexpression. **C** The tumor size and **D** the progression-free survival of Mice bearing NCI-H1975 control (blue line), PD-L1 overexpressing (red line), and PD-L1 knock-out (green line) cells were orally administered osimertinib (5 mg/kg/day), and the tumor were harvested after progression. **E** The MET CNV of NCI-H1975 control (blue bar), PD-L1 overexpressing (red bar), and PD-L1 knock-out (green bar) cells from the harvested tumor after progression. *ns* non-significance; **, *P* < 0.01

Knowing that PD-L1 can upregulate the phosphorylation of c-MET via suppressing PTPs in *EGFR*-mutant NSCLC cells, we wanted to explore the role of PD-L1 overexpression and c-MET phosphorylation in EGFR-TKI resistance. We first evaluated the difference between NCI-H1975 parental cells and NCI-H1975 PD-L1-OE cells on osimertinib sensitivity and *MET* gene copy number variation (CNV). Interestingly, there were no significant differences in osimertinib sensitivity (Fig. 5A), MET CNV (Fig. 5B), or downstream signaling responses (phospho-AKT and phospho-ERK levels under osimertinib treatment; Supplementary Fig. 7) between NCI-H1975 cells with or without PD-L1 overexpression at baseline. To further assess the impact of PD-L1 overexpression on osimertinib resistance, xenograft mouse models were established. NCI-H1975 parental cells, NCI-H1975 PD-L1-KO cells, and NCI-H1975 PD-L1-OE cells were subcutaneously implanted and treated with osimertinib through oral administration until the development of osimertinib resistance (Fig. 5C). The progression-free survival of NCI-H1975 PD-L1-OE group was significantly shorter than NCI-H1975 parental and PD-L1-KO groups (Fig. 5D, P value = 0.018). After the tumors were harvested at the time of osimertinib resistance, the *MET* CNV were significantly increased compared to baseline in NCI-H1975 PD-L1-OE group, while *MET* CNV remained unchanged in both NCI-H1975 parental and PD-L1-OE groups (Fig. 5E). Taken together, these in vivo results indicated that PD-L1 overexpression altered resistance pattern and predisposed tumor cells to develop acquired *MET* amplification under osimertinib treatment.

## Discussion

Our study provides strong evidence that PD-L1, by suppressing the activity of PTPs, leads to the upregulation of c-MET phosphorylation in human *EGFR*-mutant NSCLC cells. Importantly, we observe that *EGFR*-mutant NSCLC cells with PD-L1 overexpression are susceptible to acquiring MET amplification in response to osimertinib treatment. These findings demonstrate how the immune checkpoint PD-L1 acts unconventionally as a regulator of c-MET phosphorylation, contributing to the development of MET-dependent resistance during osimertinib therapy in *EGFR*-mutant NSCLC.

To the best of our knowledge, our study is the first to mechanistically demonstrate that c-MET can be regulated by PD-L1 using *EGFR*-mutant NSCLC cell lines. Our study showed that this regulation occurs intracellularly through association with PTP. Similarly, in a triple-negative breast cancer cell line, PD-L1 was shown to be able to directly contact with PTP to activate p38-MAPK pathway [30]. In addition, our results suggest that PD-L1 can also regulate PTPs through the suppression of mRNA expression. We further indicate that in *EGFR* mutant NSCLC, PD-L1 associates with not only one PTP but an extensive group of PTP enzymes to regulate c-MET phosphorylation. Notably, PTPs are recognized for their promiscuity due to highly-conserved active sites which selectively bind to phospho-tyrosine residues. Previous studies have shown that inhibition of one specific PTP often results in compensatory upregulation of other PTPs [31–33]. For example, PTPs have been shown to play redundant roles in insulin receptor regulation and in DNA double-strand break repair [32, 33]. Our study corroborates these findings, as we observed that the knock-down of several PTPs all leads to partial restoration of c-MET phosphorylation. This result suggests a redundancy in the c-MET dephosphorylation process and points to the presence of potential compensatory mechanisms among PTPs in *EGFR*-mutant NSCLC cells.

*MET* amplification is the most common resistance mechanism to osimertinib therapy and can be effectively targeted by combination treatment with c-MET inhibitors such as tepotinib or capmatinib [34–39]. Beyond EGFR-mutant NSCLC, *MET* amplification has also been reported as a mechanism of resistance in other oncogene-driven NSCLCs, including those with ALK fusions or KRAS G12C mutations. However, it occurs less frequently in these contexts compared to EGFR-mutant NSCLC [40, 41]. Whether the PD-L1-dependent *MET* regulation observed in our EGFR-mutant NSCLC model also plays a role in other oncogene-driven subtypes remains unexplored and should be separately investigated in the future study.

While the exact molecular mechanism underlying gene amplification are still poorly understood, some cytogenetic events that result from chromosomal instability such as breakage-fusion bridge cycle and the formation of double minute chromosomes have been proposed to be important steps leading to gene amplification [42–44]. Our study revealed that in *EGFR*-mutant NSCLC cells (NCI-H1975) overexpressing PD-L1, several DNA repair-associated genes that are related to genomic instability were upregulated, such as SWI5, ARRB2, SMARCA5, XRCC1, CCDC88A, MCM2, CENPX, POLE3, and RUVBL1 (Supplementary Fig. 8). For example, SWI5 plays a critical role in homologous recombination and TKI resistance [45, 46], while SMARCA5 is involved in chromatin remodeling and frequently deregulated in cancers [47, 48]. Similarly, CENPX ensures proper chromosome segregation [49], and RUVBL1 participates in DNA repair, transcription, and snoRNP assembly [50]. Interestingly, *MET* amplification was not found in our PD-L1 overexpressing cells before osimertinib treatment, and it also rarely occurs in treatment-naïve *EGFR*-mutant NSCLC patients [24]. Therefore, survival stress imposed by EGFR-TKI treatment is crucial to drive MET amplification.

Recently, MARIPOSA trial has demonstrated that combining lazertinib with amivantamab to inhibit both EGFR and c-MET signaling pathways in the first-line setting significantly improves both progression-free and overall survival, but at a cost of increased toxicities [51]. Therefore, predictive biomarkers are urgently needed to identify patient population that would benefit the most from combination treatment. Our study provides preclinical evidence suggesting that PD-L1 may be a candidate predictive biomarker identifying patients at risk of acquiring *MET* amplification, who might therefore benefit more from upfront c-MET inhibition.

In addition to phosphatase-mediated regulation, multiple mechanisms are known to influence c-MET phosphorylation, including ligand binding (e.g., HGF), receptor dimerization, membrane localization, RTK crosstalk, and protein degradation [52]. In our study, we systematically ruled out HGF as a significant factor, as *EGFR*-mutant NSCLC cells expressed negligible HGF at baseline, and PD-L1 overexpression or knockout did not significantly alter secreted HGF levels (Supplementary Fig. 5). This supports a ligand-independent mechanism of MET activation in our model. Regarding receptor dimerization and RTK crosstalk, c-MET is known to heterodimerize with other RTKs, such as HER3 [53], in lung cancer. Whether PD-L1 expression influences HER3–c-MET interactions, thereby modulating c-MET phosphorylation through RTK crosstalk, is an intriguing possibility that warrants further investigation. Notably, no changes in total MET protein levels were observed in our experiments, arguing against regulation via protein degradation. Collectively, our data and complementary database analyses support that PD-L1 regulates c-MET phosphorylation primarily via suppression of PTP activity, while additional regulatory pathways may also contribute and deserve continued investigation.

There are some limitations in this study. First, the study validated the relationship between PD-L1 expression and c-MET phosphorylation in several *EGFR*-mutant cell lines. However, CRISPR knock-out experiments were only carried out using NCI-H1975 cells. The results may not be fully generalizable to other cell lines, which were validated using siRNA knock-down technology. Second, several PTPs were identified as potential targets of PD-L1 regulation in our study. However, we could not further determine if certain PTPs are more crucial than others in this PD-L1-PTPs regulatory pathway. In addition, it should be acknowledged that in vitro PTP assays cannot fully recapitulate the activation status of cellular PTPs, as the catalytic cysteine residues are highly sensitive to redox changes [54]. Cell disruption and altered redox states may significantly affect PTP activity, thereby limiting the interpretation of these assays. Lastly, our in vivo experiments on osimertinib resistance indicates that tumor cells with PD-L1 overexpression and high c-MET phosphorylation before osimertinib treatment were predisposed to acquire *MET* amplification at the time of osimertinib resistance. However, we can not exclude the possibility that other mechanisms, such as EGFR, HER3 overexpression, or bypass activation of other canonical signaling pathways may also lead to the development of *MET* amplification in clinical settings.

## Conclusions

The current work expands our understanding of PD-L1’s function beyond its role as an immune checkpoint in *EGFR*-mutant NSCLC. We have uncovered how PD-L1 regulates c-MET phosphorylation and how it may contribute to *MET* amplification under osimertinib treatment. Our findings highlight PD-L1’s tumor-intrinsic role in influencing tumor evolution patterns and resistance to targeted therapy in *EGFR*-mutant NSCLC.

## Supplementary Information

Supplementary material 1. Fig. 1. The effects of different concentration of serum on the PD-L1 expression in NCI-H1975 with or without PD-L1 overexpression. Western blot analysis of PD-L1 expressions after incubation with different concentrations of serum (0, 0.5, 1, 3, 5, and 10%) for 24 h in NCI-H1975 with or without PD-L1 overexpression. OE, overexpression. Fig. 2. Validation of PD-L1 knock-out in NCI-H1975 cells. (A and B) The sequences and activities of each sgRNA against PD-L1. (C) Endogenous PD-L1 and (D) induced PD-L1 by IFNγ administration (100 ng/mL) for 24 h, and the knock-out of PD-L1 in NCI-H1975 cells was verified by Western blot analysis using antibodies against PD-L1 and α-tubulin. 35. Fig. 3. Effects of PD-L1 knock-down on c-MET phosphorylation in NCI-H1975 cells. Western blot analysis of phospho-c-MET expressions after knock-down of PD-L1 using siRNA technology for 40 h in NCI-H1975 cells. Fig. 4. PD-L1 blockers alone have no effect on survival or phosphor-c-MET expression in NCI-H1975 PD-L1 OE cells. The sensitivity to (A) durvalumab and (B) atezolizumab treatment in NCI-H1975 PD-L1 OE cells. Western blot analysis of phospho-c-MET expressions in NCI-H1975 PD-L1 OE cells treated with (C) durvalumab and (D) atezolizumab in dose-dependent experiments (lane 1-6 for 0, 6.25, 12.5, 25, 50, 100 μg/mL PD-L1 blocker for 24 h). Fig. 5. Basal and experimental analysis of HGF expression in human LUAD cells. (A) Basal HGF gene expression levels among human LUAD cell lines obtained from the EMBL-EBI Expression Atlas database. Green and white boxes indicate EGFR-mutant and EGFR wild-type LUAD cell lines, respectively. (B) Standard curve for human HGF ELISA used in quantification. (C) Secreted HGF protein levels measured by ELISA in H1975 control, PD-L1- 36 overexpressing (OE), PD-L1 knockout (KO), and HGF-overexpressing cells (positive control). Fig. 6. Overexpression of PD-L1 suppressed the enzymatic activity of protein tyrosine phosphatases in EGFR-mutant NSCLC cells. Whole cell extracts were harvested from NCI-H1975 cells with or without PD-L1 overexpression (PD-L1 OE), and the enzymatic activity of PTPs was measured. (blue line for control, and red line for PD-L1 OE). Fig. 7. PD-L1 overexpression did not significantly alter the downstream AKT or ERK phosphorylation response to osimertinib. NCI-H1975 cells with or without PD-L1 overexpression (PD-L1 OE) were treated with osimertinib (25 nM) for 24 h, and whole-cell lysates were analyzed by Western blot for phospho-AKT and phospho-ERK expression. Fig. 8. Gene signature reveals DNA associated functions contributed by PD-L1 in EGFR-mutant NSCLC cells. The circos plots represent the significant functions and genes involved in DNA-associated 37 functions which enriched in NCI-H1975 PD-L1 OE cells.

Supplementary material 2. Information for cherry-pick custom library siRNAs against PTPs.

## Data Availability

No datasets were generated or analysed during the current study.
