# Supplementary material for: PD-L1 regulates c-MET phosphorylation and contributes to MET-dependent resistance to osimertinib in EGFR-mutant NSCLC
Source: J Biomed Sci. 2025 Oct 9;32:94. doi: 10.1186/s12929-025-01181-3 (PMC12509346; doi:10.1186/s12929-025-01181-3)
Supplement: Supplementary file 1 — Supplementary material 1. Fig. 1. The effects of different concentration of serum on the PD-L1 expression in NCI-H1975 with or without PD-L1 overexpression. Western blot analysis of PD-L1 expressions after incubation with different concentrations of serum (0, 0.5, 1, 3, 5, and 10%) for 24 h in NCI-H1975 with or without PD-L1 overexpression. OE, overexpression. Fig. 2. Validation of PD-L1 knock-out in NCI-H1975 cells. (A and B) The sequences and activities of each sgRNA against PD-L1. (C) Endogenous PD-L1 and (D) induced PD-L1 by IFNγ administration (100 ng/mL) for 24 h, and the knock-out of PD-L1 in NCI-H1975 cells was verified by Western blot analysis using antibodies against PD-L1 and α-tubulin. 35. Fig. 3. Effects of PD-L1 knock-down on c-MET phosphorylation in NCI-H1975 cells. Western blot analysis of phospho-c-MET expressions after knock-down of PD-L1 using siRNA technology for 40 h in NCI-H1975 cells. Fig. 4. PD-L1 blockers alone have no effect on survival or phosphor-c-MET expression in NCI-H1975 PD-L1 OE cells. The sensitivity to (A) durvalumab and (B) atezolizumab treatment in NCI-H1975 PD-L1 OE cells. Western blot analysis of phospho-c-MET expressions in NCI-H1975 PD-L1 OE cells treated with (C) durvalumab and (D) atezolizumab in dose-dependent experiments (lane 1-6 for 0, 6.25, 12.5, 25, 50, 100 μg/mL PD-L1 blocker for 24 h). Fig. 5. Basal and experimental analysis of HGF expression in human LUAD cells. (A) Basal HGF gene expression levels among human LUAD cell lines obtained from the EMBL-EBI Expression Atlas database. Green and white boxes indicate EGFR-mutant and EGFR wild-type LUAD cell lines, respectively. (B) Standard curve for human HGF ELISA used in quantification. (C) Secreted HGF protein levels measured by ELISA in H1975 control, PD-L1- 36 overexpressing (OE), PD-L1 knockout (KO), and HGF-overexpressing cells (positive control). Fig. 6. Overexpression of PD-L1 suppressed the enzymatic activity of protein tyrosine phosphatases in EGFR-mutant NSCL [file 12929_2025_1181_MOESM1_ESM.pdf]

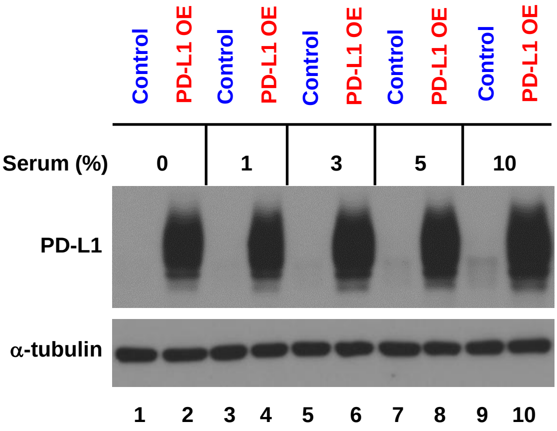
Control
PD-L1 OE
Control
PD-L1 OE
Control
PD-L1 OE
Control
PD-L1 OE
Control
PD-L1 OE
Serum (%)
0
1
3
5
10
PD-L1
α-tubulin
1
2
3
4
5
6
7
8
9
10

Supple. Fig. 1

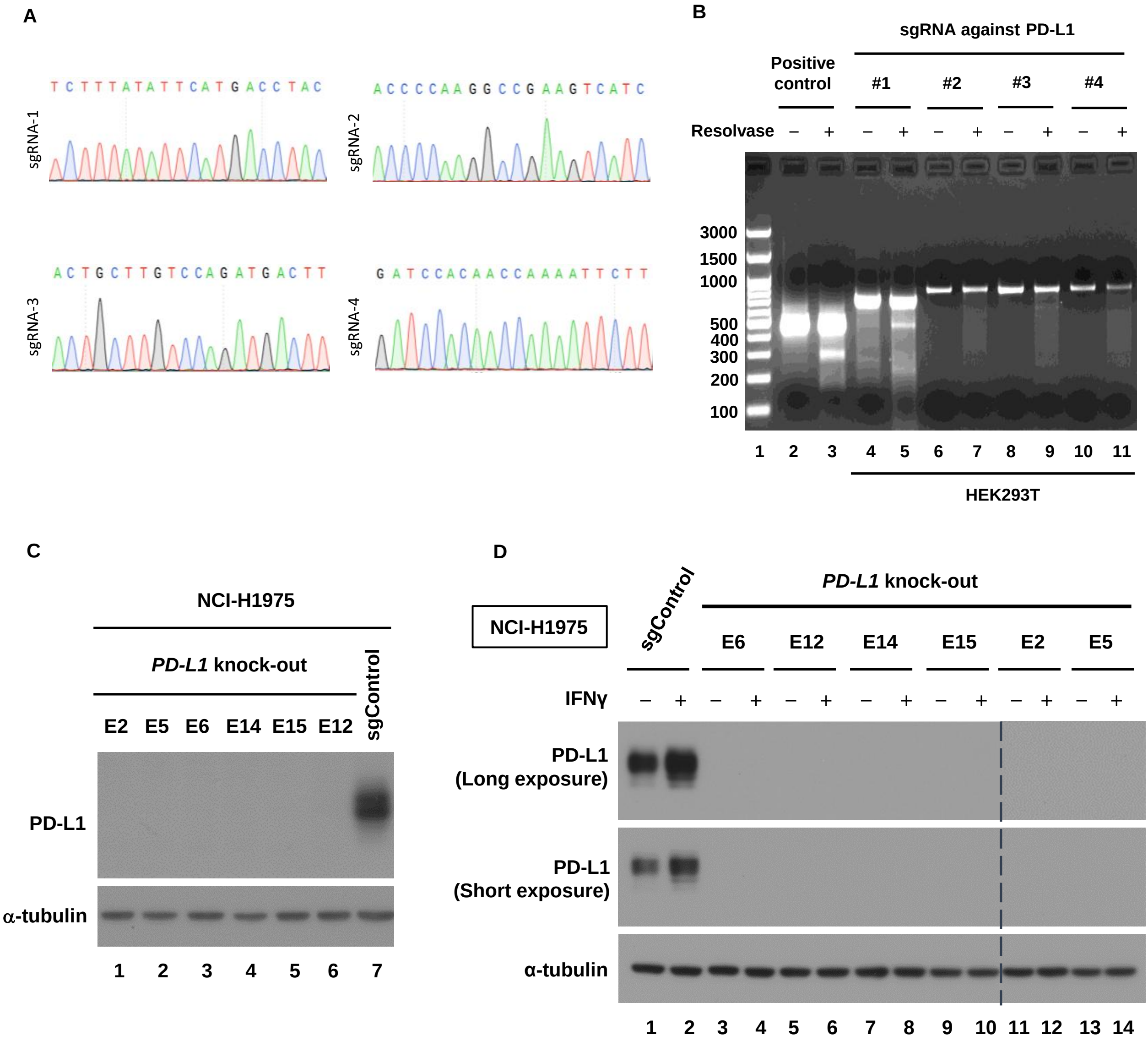
A
B
C
D
sgRNA-1
TCTTTATATTCATGACCTAC
sgRNA-2
ACCCCAAGGCCGAAGTCATC
sgRNA-3
ACTGCTTGTCCAGATGACTT
sgRNA-4
GATCCACAACCAAAATTCTT
sgRNA against PD-L1
Positive control
#1
#2
#3
#4
Resolvase
3000
1500
1000
500
400
300
200
100
1 2 3 4 5 6 7 8 9 10 11
HEK293T
NCI-H1975
PD-L1 knock-out
E2 E5 E6 E14 E15 E12
sgControl
PD-L1
α-tubulin
1 2 3 4 5 6 7
NCI-H1975
sgControl
PD-L1 knock-out
E6 E12 E14 E15 E2 E5
IFNγ
PD-L1 (Long exposure)
PD-L1 (Short exposure)
α-tubulin
1 2 3 4 5 6 7 8 9 10 11 12 13 14

Supple. Fig. 2

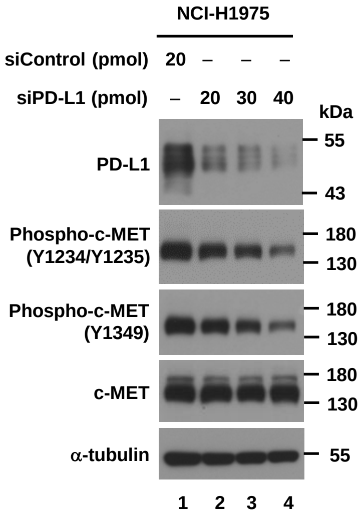
NCI-H1975
siControl (pmol) 20 – – –
siPD-L1 (pmol) – 20 30 40
kDa
PD-L1
55
43
Phospho-c-MET (Y1234/Y1235)
180
130
Phospho-c-MET (Y1349)
180
130
c-MET
180
130
α-tubulin
55
1 2 3 4

Supple. Fig. 3

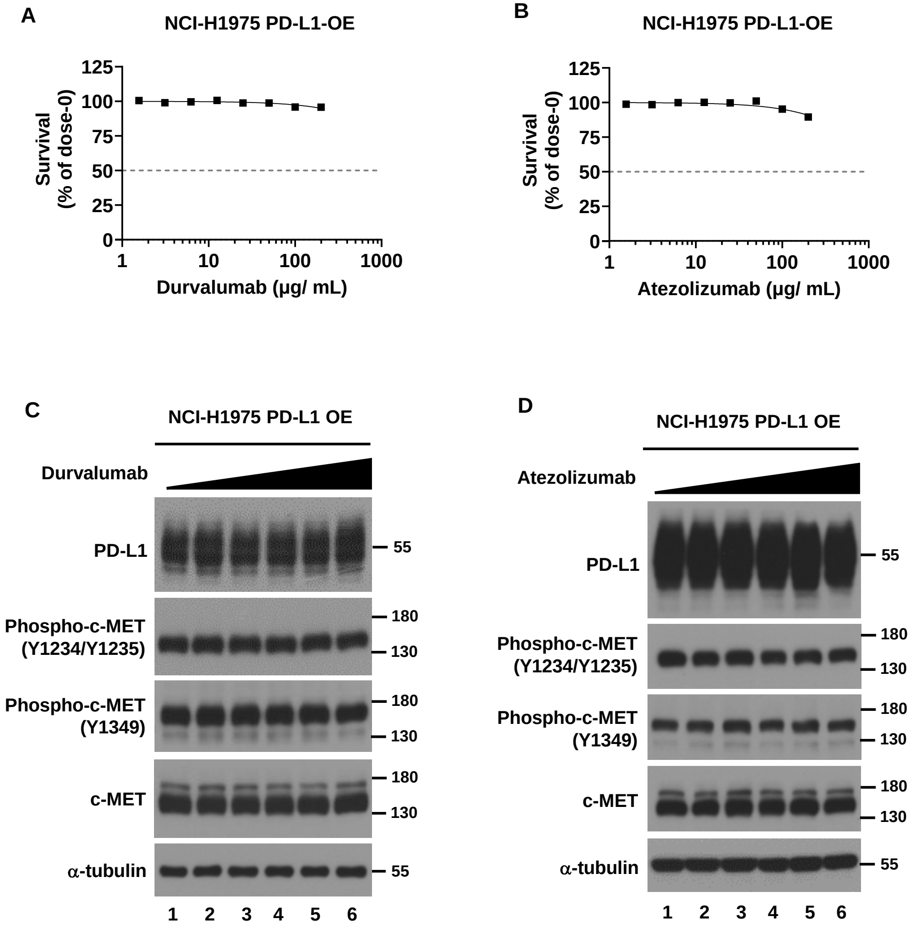

A
NCI-H1975 PD-L1-OE
Survival (% of dose-0)
125
100
75
50
25
0
1
10
100
1000
Durvalumab (μg/ mL)
B
NCI-H1975 PD-L1-OE
Survival (% of dose-0)
Atezolizumab (μg/ mL)
C
NCI-H1975 PD-L1 OE
Durvalumab
PD-L1
Phospho-c-MET (Y1234/Y1235)
Phospho-c-MET (Y1349)
c-MET
α-tubulin
55
180
130
1 2 3 4 5 6
D
NCI-H1975 PD-L1 OE
Atezolizumab

Supple. Fig. 4

A

EBC-1	ABC-1	HCC2885	LXFL529	NCI-H1703	NCI-H2073	NCI-H810	HCC78	NCI-H1770
0	0	0	0	0	0	0	0.2	1
HCC2279	CAL-12T	HCC364	MOR	NCI-H1734	NCI-H2106	RERF-LC-KJ	NCI-H1155	NCI-H1395
0	0	0	0	0	0	0	0.2	3
HCC2935	Calu-3	HCC366	NCI-H1299	NCI-H1781	NCI-H2110	RERF-LC-OK	NCI-H1573	NCI-H920
0	0	0	0	0	0	0	0.3	4
HCC4006	EKVX	HCC44	NCI-H1355	NCI-H1792	NCI-H2122	DV-90	NCI-H2172	NCI-H522
0	0	0	0	0	0	0.1	0.4	5
HCC4011	HCC15	HCC461	NCI-H1373	NCI-H1869	NCI-H2126	HCC1171	NCI-H650	VMRC-LCD
0	0	0	0	0	0	0.1	0.4	19
NCI-H1650	HCC1534	HCC515	NCI-H1568	NCI-H1915	NCI-H2135	NCI-H1651	NCI-H1838	NCI-H2023
0	0	0	0	0	0	0.1	0.6	30
NCI-H1975	HCC193	HOP-62	NCI-H1623	NCI-H1944	NCI-H2228	NCI-H1793	NCI-H1437	RERF-LC-MS
0	0	0	0	0	0	0.1	0.7	130
NCI-H820	HCC2270	KNS-62	NCI-H1648	NCI-H2009	NCI-H2347	NCI-H358	COLO 699	NCI-H1581
0	0	0	0	0	0	0.1	1	144
HCC827	HCC2302	LXF-289	NCI-H1693	NCI-H2030	NCI-H322T	SW 900		
3	0	0	0	0	0	0.1	0	144

EGFR mutant LUAD lines   *EGFR* wild-type LUAD lines

B

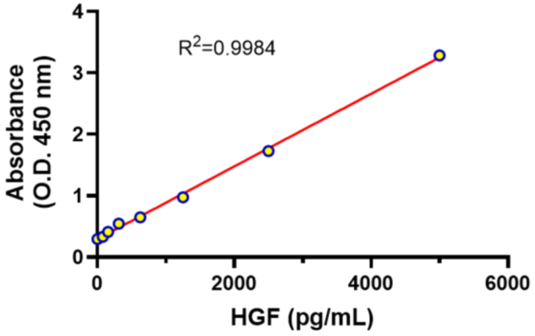

C

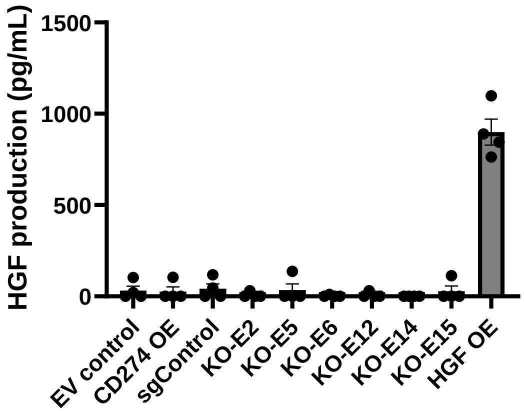

Supple. Fig. 5

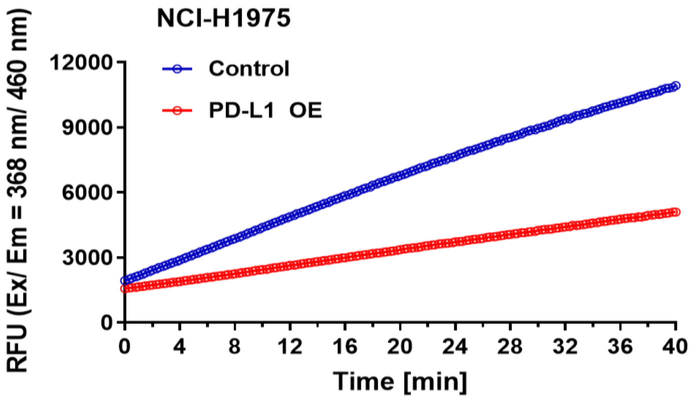

NCI-H1975
RFU (Ex/ Em = 368 nm/ 460 nm)
12000
9000
6000
3000
0
Control
PD-L1 OE
0
4
8
12
16
20
24
28
32
36
40
Time [min]

Supple. Fig. 6

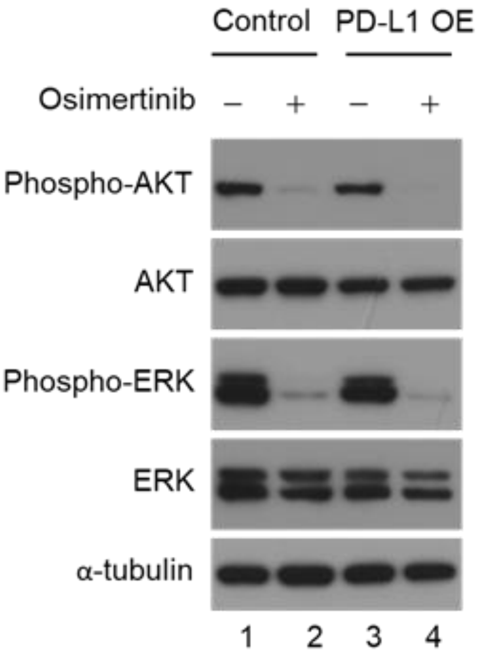
Control
PD-L1 OE
Osimertinib
–
+
–
+
Phospho-AKT
AKT
Phospho-ERK
ERK
α-tubulin
1
2
3
4

Supple. Fig. 7

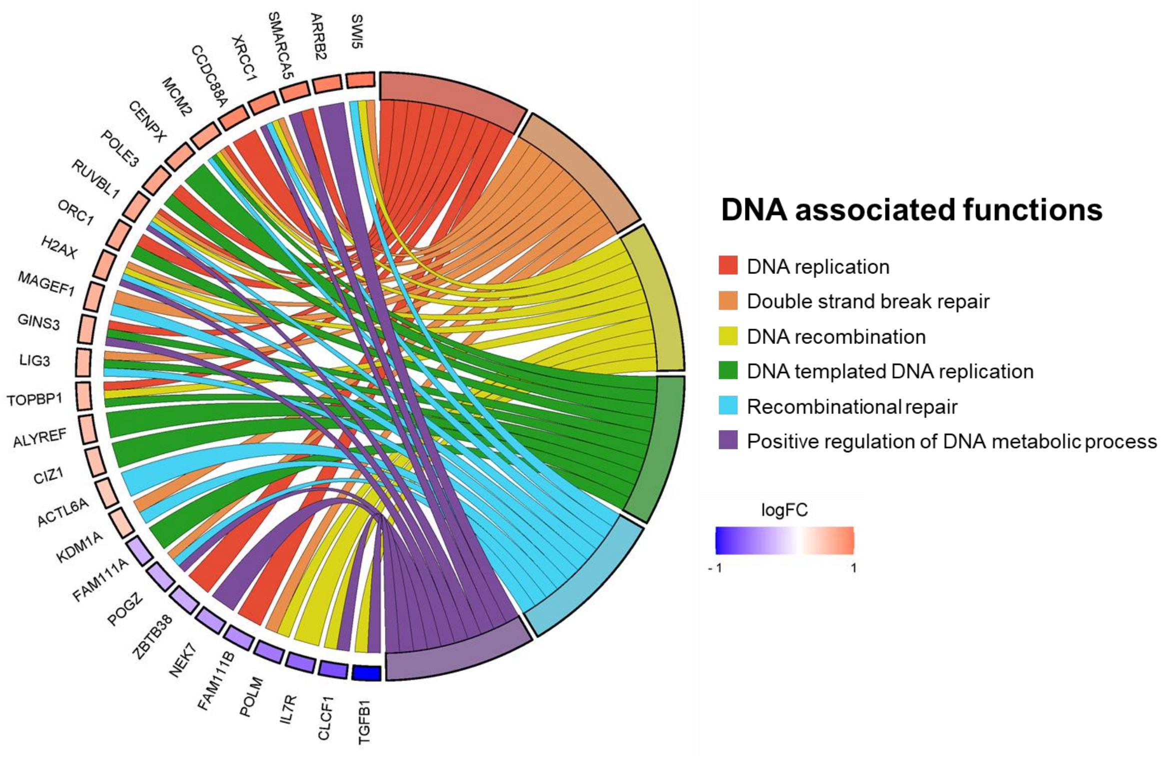
SWI5
ARRB2
SMARCA5
XRCC1
CCDC88A
MCM2
CENPX
POLE3
RUVBL1
ORC1
H2AX
MAGEF1
GINS3
LIG3
TOPBP1
ALYREF
CIZ1
ACTL6A
KDM1A
FAM111A
POGZ
ZBTB38
NEK7
FAM111B
POLM
IL7R
CLCF1
TGFB1
DNA associated functions
DNA replication
Double strand break repair
DNA recombination
DNA templated DNA replication
Recombinational repair
Positive regulation of DNA metabolic process
logFC
- 1
1

Supple. Fig. 8
